# Kelvin-Helmholtz instability in a compressible dust fluid flow

**DOI:** 10.1038/s41598-023-30992-3

**Published:** 2023-03-09

**Authors:** Krishan Kumar, P. Bandyopadhyay, Swarnima Singh, Vikram S. Dharodi, A. Sen

**Affiliations:** 1 Institute For Plasma Research, A CI of Homi Bhabha National Institute, Bhat, Gandhinagar, Gujarat 382428 India; 2 Physics Department, Auburn University, Auburn, AL 36849 USA

**Keywords:** Physics, Plasma physics

## Abstract

We report the first experimental observations of a single-mode Kelvin-Helmholtz instability in a flowing dusty plasma in which the flow is compressible in nature. The experiments are performed in an inverted $\Pi$-shaped dusty plasma experimental device in a DC glow discharge Argon plasma environment. A gas pulse valve is installed in the experimental chamber to initiate directional motion to a particular dust layer. The shear generated at the interface of the moving and stationary layers leads to the excitation of the Kelvin-Helmholtz instability giving rise to a vortex structure at the interface. The growth rate of the instability is seen to decrease with an increase in the gas flow velocity in the valve and the concomitant increase in the compressibility of the dust flow. The shear velocity is further increased by making the stationary layer to flow in an opposite direction. The magnitude of the vorticity is seen to become stronger while the vortex becomes smaller with such an increase of the shear velocity. A molecular dynamics simulation provides good theoretical support to the experimental findings.

## Introduction

The Kelvin-Helmholtz (KH) is a hydrodynamic instability that occurs at the interface of shearing fluids^1,2^. It manifests itself as the growth of a boundary distortion that causes the fluid to flow from one side of the interface to the other side that eventually results in the formation of a nonlinear vortex structure at the interface. The KH instability is ubiquitous in both natural and laboratory systems. It can be seen in surface waves generated by a wind blowing over the ocean or a river^3–6^, during air flowing through clouds^7–9^, in various stellar processes^10,11^, such as astrophysical jets^12–14^, protoplanetary disks^15^, magnetospheric boundaries^16^, *etc.*. The KH instability can emerge at the interface of both incompressible^17–20^ and compressible^21–25^ flowing fluids.

The KH instability-induced turbulence at the interface of sheared plasma flow transports mass, momentum, and energy across the boundary which helps to understand a variety of astrophysical phenomena^26,27^. Other than astrophysical situations, the KH instability has also been extensively studied in flowing magnetized plasmas^28–31^. In some other experiments, a novel technique was employed using a laser to initiate the KH instability in high energy density plasmas^32,33^. In astrophysical fluids like protoplanetary disks, cometary outflows and tori around supermassive black holes, there exist a large dust component and it is important to investigate the KH instability in such plasmas. A dusty (or complex) plasma medium is a collection of ions, electrons, neutrals, and highly charged massive dust grains and is a useful model to represent such plasmas. The addition of these micron or sub-micron particles in the conventional plasma adds richness in the collective dynamics of the system. Depending upon the ratio of electrostatic energy to the thermal energy of the particles (defined as the Coulomb coupling parameter, $\Gamma$), a complex plasma medium can be in a solid, a liquid or gaseous state^34–36^.

Numerous theoretical^37–39^ and simulation studies^40–46^ have been carried out to explore the KH instability in a dusty plasma. N. D’Angelo *et al.* theoretically studied the KH instability in a magnetized dusty plasma for negatively and positively charged dust grains. Ashwin *et al.* investigated the KH instability in two-dimensional strongly coupled Yukawa liquids with the help of molecular dynamics simulations^40^. They showed that the growth rate of the instability increases with the increase of the Coulomb coupling parameter and a vortex roll forms in the non-linear regime. Sanat *et al.* also reported the KH instability for both weakly^41^ and strongly^43^ coupled dusty plasmas. However, to the best of our knowledge, a definitive laboratory observation of the KH instability in a dusty plasma system has not been reported so far.

In the past, several experiments have been devoted to the generation of sheared flows in a dusty plasma medium^47–52^ but have failed to observe the KH instability at the interface of the shearing fluids. The reasons could be insufficient dust density and/or subcritical shear velocities. In this paper, we experimentally demonstrate the existence of the KH instability in a compressible dust fluid flow. A fluid is said to be compressible when the Mach number of the flow is close to the value of ‘1’ or greater than ‘1’. In such a regime the speed of sound in the incompressible fluid is large enough so that there is no density variation in space and time. A gas pulse valve is employed in our experiments to initiate a directional flow in a particular layer of the dust fluid with a speed nearly equal to or higher than the dust acoustic speed. The average dust fluid flow velocity is controlled by altering the difference between the background plasma pressure and the inlet pressure of the pulsed valve. A single-mode KH instability emerges at the interface of the moving and stationary layers and a vortex forms at the interface. It is observed that the vortex size becomes smaller with an increase in the inlet gas pressure of the pulsed valve. The shear velocity increases even further when the stationary layer is made to flow in the opposite direction, which results in the enhancement of the magnitude of vorticity and a reduction in the size of the KH vortex. A molecular dynamics simulation is also performed and its findings are seen to compare favourably with the experimental observations.

The paper is organized as follows: the experimental apparatus and details of the experimental procedure are described in Sect. “Experimental apparatus and procedure”. Section “Experimental results and discussion” contains the experimental results on the generation of the Kelvin-Helmholtz instability and a comprehensive discussion on it. Section “Molecular dynamic simulation” presents the results of a molecular dynamic simulation and compares them to the experimental findings. Section “Conclusion” provides a summary and some concluding remarks.

## Experimental apparatus and procedure

**Figure 1:**
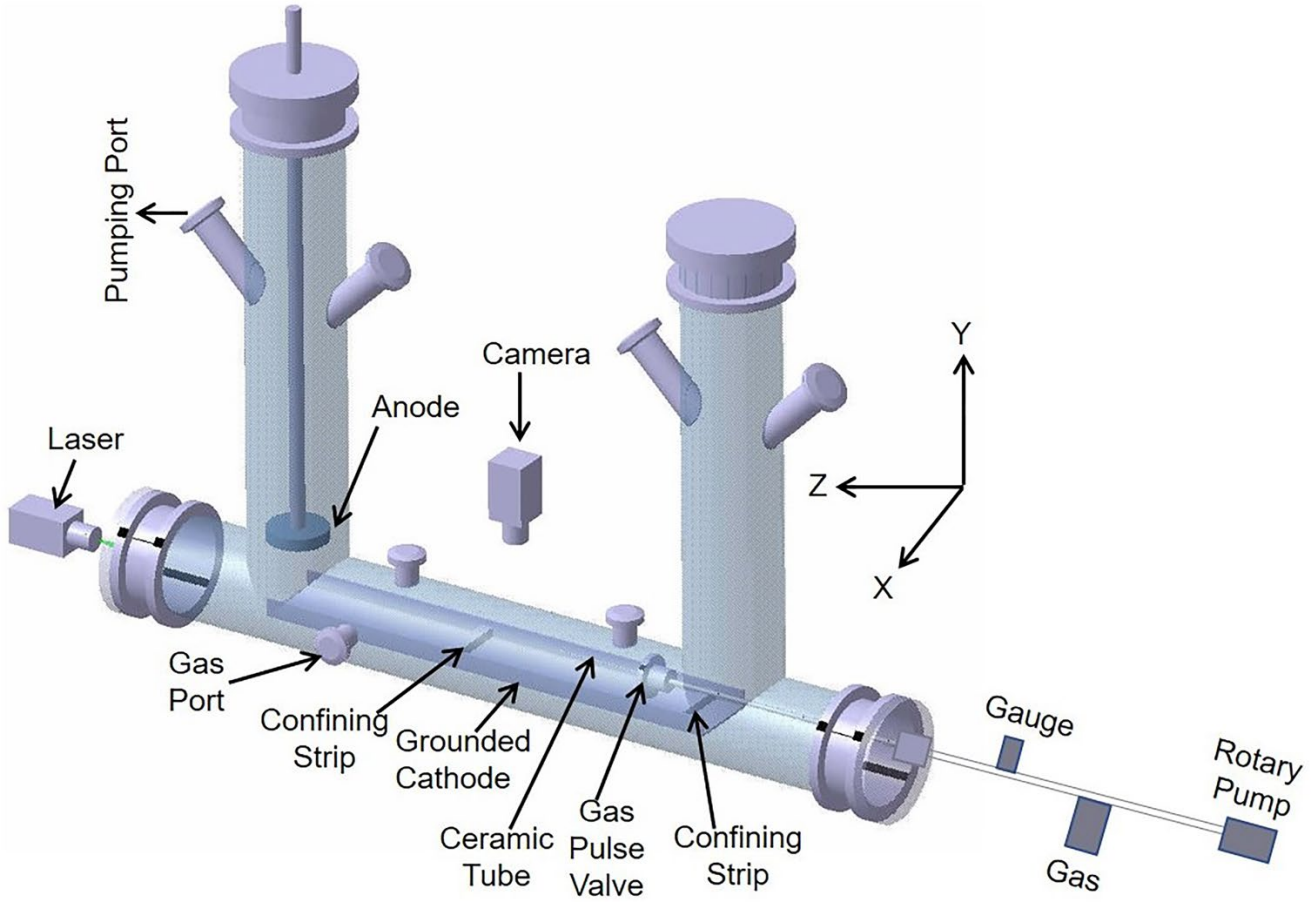
(**a**) A schematic diagram of the dusty plasma experimental (DPEx) device.

The experiments associated with the present study are conducted in the Dusty Plasma Experimental (DPEx) device whose schematic diagram is given in Fig. 1. For a more detailed description of the set-up along with its diagnostics the reader is referred to Ref. ^53^. A rotary pump is connected to one of the radial ports of the experimental chamber to attain a base pressure of 0.1 Pa and a mass flow controller is employed to feed Argon gas in the chamber to achieve working pressures in the range of 10–12 Pa. A Pirani gauge is attached to record the pressure in the experimental chamber. The aforementioned inverted $\Pi$-shaped device has a circular shaped anode and a long tray-shaped grounded cathode used for the production of a DC glow discharge plasma. Micron-sized Kaolin (aluminum silicate) dust particles are sprinkled on the cathode between the two rectangular stainless steel (SS) strips, which are kept approximately 30 cms apart (see Fig. 1). These strips are used to confine the dust particles axially. The radial confinement of the dust particles is provided by the bent edges of the cathode. To generate the sub-sonic to super-sonic flows in a particular dust layer a gas pulse valve having an orifice diameter of 0.5 mm is connected with a SS pipe of 3.5 mm inner diameter. A rotatory pump and a gas dosing valve are attached with the pulse valve to maintain the desired Argon gas pressure at the input side of the gas pulse valve. A Pirani gauge is also connected to monitor the gas pressure as shown in Fig. 1. A ceramic tube of a length of 10 cm and an inner diameter of 0.5 mm is attached to the output of the gas pulse valve to initiate a directional motion of the dust particles in a particular dust layer, which also hinders unwanted arcing. This complete assembly is installed in one of the axial ports of the chamber with the help of a Wilson feed-through.

A rarefied Argon environment of 10–12 Pa is created in the chamber and an Argon plasma is generated by applying a DC voltage of 300–380 V between the asymmetric electrodes. Plasma parameters like electron temperature, $T_{e} \sim 2-5$ eV and plasma density, $n_{i} \sim 0.5-3 \times 10^{15}$ $m^{-3}$ are measured using a single Langmuir probe over a wide range of aforementioned discharge parameters^53^. The dust particles acquire a negative charge in the plasma environment by absorbing more electrons than ions and levitate at the cathode sheath region between the two SS rectangular strips by balancing the upward electrostatic force with the downward gravitational force. A green laser of wavelength 532 nm and power of 100 mW is used for illuminating the levitated dust particles and their dynamics are captured using a CCD camera. The recorded sequences of images are then analyzed with the help of a MATLAB-based open-access Particle Image Velocimetry (PIV) code^54^.

**Figure 2:**
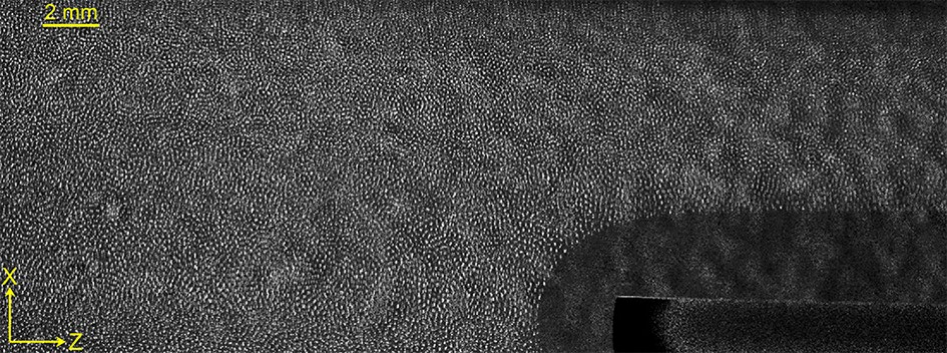
Snapshot of equilibrium dust cloud.

For the present set of experiments, the set-up is operated at a particular discharge voltage of 350 V and neutral gas pressure of 11 Pa. Fig. 2 shows the typical camera image of the equilibrium dust cloud at that particular discharge condition. As shown in the bottom right corner of Fig. 2, a dust-free region (void) is formed due to the presence of space charged region (sheath) around the ceramic tube. It is worth mentioning that the dust particles in the equilibrium dust cloud do not show any directional motion; instead they exhibit only random thermal agitation. At this specific discharge condition, the dusty plasma parameters like inter-particle distance (a), average particle diameter (d), and dust Debye length ($\lambda _{D}$) are estimated to be 160 $\mu$m, 3 $\mu$m, and 40 $\mu$m, respectively. The Collision Enhanced Collection (CEC) model^55^ is used to estimate the average charge on a dust particle ($Q_{d}$) which yields a value of $\sim 6.1 \times 10^{3}$e. The Coulomb coupling parameter for these plasma and dusty plasma parameters is estimated using the expression $\Gamma =(Q_{d}^{2}/4\pi \varepsilon _{0}ak_{B}T_{d})exp(-{a}/\lambda _{D})$^34,56,57^ and comes out to be 30. For the screening parameter of $\kappa =a/\lambda _{D}=4$, $\Gamma$ value essentially ensures that the dusty plasma in the present discharge condition remains in the fluid regime^56^.

To generate a flow in the equilibrium dust fluid, the input Argon gas pressure of the gas pulse valve is set over the range of 200–400 Pa, which is much higher than the background pressure of the chamber. The neutral particles come out and rush to the chamber from the pulse valve to equilibrate the gas pressure during the opening time ($\sim 10$ s) of the pulse valve. As a result, a strong neutral drag force acts on the dust particles, which causes the particles to flow toward the anode. It is to be noted that, the velocity of the dust layer depends on the pressure difference between the pulse valve and the chamber. To increase the shear flow in the dust fluid, two dust layers are made to flow in opposite directions by using a gas pulse valve and single gas injection technique^58^. The gas pulse valve is employed to make the flow from right to left whereas the single gas injection technique is used to make the flow of another layer in the dust fluid from left to right.

**Figure 3:**
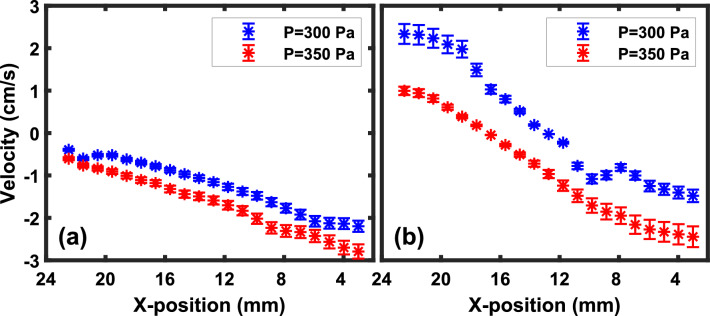
Variation of velocities for (**a**) single layer flow and (**b**) double layer flow, of the dust fluid along X-direction for a particular Z-position of 10 mm. Here, the dust fluid flows along the Z-direction.

Figure 3 shows the variation of velocity of dust fluid along the X-position at a particular Z-position of 10 mm for a single layer flow (see Fig. 3a) and a double layer flow (see Fig. 3b). In both cases, the dust fluid flows along the Z-direction. In the case of a single-layer flow, the dust fluid remains almost stationary at the top and then it moves with gradual increasing velocity and becomes maximum at $X \sim 4$ mm. However, in the case of double-layer flow, both the layers move in opposite directions (as velocity changes its sign) creating a larger velocity shear in the dusty plasma medium. It is also to be noted that in both cases, the magnitude of the dust fluid flow velocity increases with the increase of the pressure difference. A quantitative variation of the flow velocities with the gas pressure difference for a single-layer flow is provided below.

**Figure 4:**
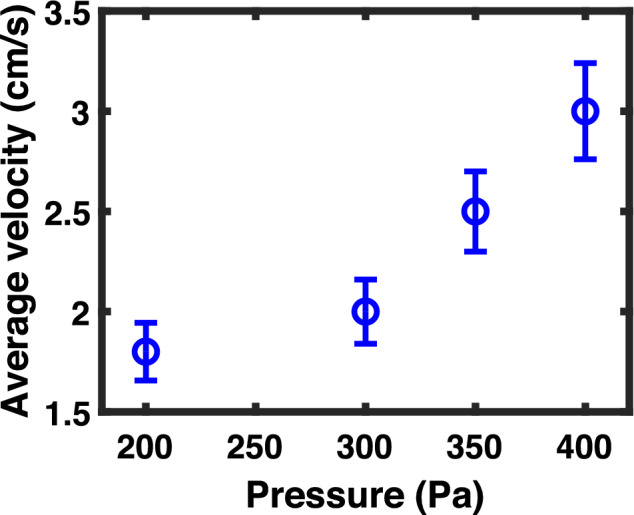
Variation of the average velocity of dust layer with the input pressure of pulse valve at a constant chamber pressure of 11 Pa.

To examine the dependence of the fluid velocity on the pressure difference, a controlled experiment is performed by changing the gas pressure of the pulse valve and maintaining the constant pressure in the chamber. Fig. 4 shows the variation of the average velocity of dust fluid with the input pressure of the pulse valve at a constant pressure of 11 Pa in the chamber. The velocity is averaged out along Z-position at a particular X-position of 15 mm. It is observed that the average velocity of dust fluid increases with the increase in the inlet pressure of the gas pulse valve. A detailed investigation is carried out, which is discussed later in the next section, to understand how the dynamics of the vortex structure get modified with the inlet pressure of the pulse valve.

To examine the compressibility of this flowing dust fluid, we have estimated as well as measured the phase velocity of dust acoustic waves (DAW). The phase velocity of DAW for the above experimental parameters is estimated from the expression, $C_d =Z_d\sqrt{n_dKT_i/m_dn_i}$, where $n_d\sim 6\times 10^{10}$ $m^{-3}$ is the dust density and $m_d \sim 3.75\times 10^{-14}$ Kg is the average mass of the dust particles. For these parameters, the phase velocity turns out to be $1.7\pm 0.2$ cm/s. An experiment is also carried out to excite dust acoustic waves by applying a sinusoidal pulse to a wire mounted on the cathode, with an amplitude and frequency of 40 V and 2 Hz, respectively. It is found that the dust acoustic wave propagates in the dust fluid with a velocity of $\sim 2.1\pm 0.1$ cm/s which is close to the estimated value of the phase velocity for the experimental condition. A knowledge of the phase velocity of the acoustic wave and the flow velocity of the dust fluid allows us to determine whether the fluid flow is compressible or incompressible. The Mach number, *M* (the ratio of fluid flow velocity to the phase velocity of DAW) becomes close to one or more in our experiments, hence we can safely assume that the dust fluid flow for our experimental conditions remains compressible^59^.

## Experimental results and discussion

**Figure 5:**
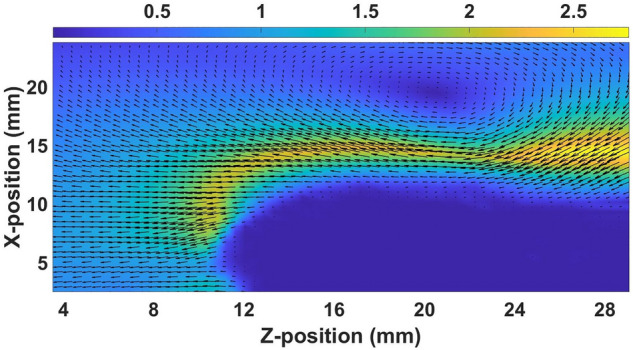
The velocity vector field with the magnitude of the velocity (cm/s) for the single layer flow at the input pressure of the pulse valve is 300 Pa.

The KH instability emerges at the interface of the shear layers of the dust fluid when the shear velocity crosses the threshold value. In the first set of experiments, the shear in the equilibrium dust fluid is generated by using a gas pulse valve as discussed in Sect. “Experimental apparatus and procedure”. The dynamics of the particles in the equilibrium dust cloud change significantly when the pulse valve is instantaneously opened for a few seconds. Fig. 5 depicts the color map of the magnitude and vector direction of the velocity field of the dust fluid for an inlet gas pressure of the pulse valve of 300 Pa. It is seen in the figure that the bottom layer of the dust fluid moves with a larger velocity ($\sim$ 2 cm/s) as compared to the top layer, which causes a shear at the interface of the two layers. This shear flow at the interface is responsible for the emergence of KH instability in the medium, which leads to the formation of a single vortex at *Z* and *X* positions of 20 mm and 20 mm, respectively. It is to be noted that the center of the single-mode KH vortex and its dimension change with the difference in gas pressure.

The growth rate for the KH instability in a compressible fluid is estimated from the expression^59^,

1 $$\begin{aligned} \gamma =\frac{K U}{2}\sqrt{1-A^{2}}\left( \frac{\sqrt{-1-M^{2}+\sqrt{1+4M^{2}}}}{M}\right) . \end{aligned}$$

The wave number of the modulation is represented by *K*, whereas *U* indicates the velocity difference at the interface. The Atwood number, *A* can be expressed by $(\rho _{2}-\rho _{1})/(\rho _{2}+\rho _{1})$ where, $\rho _{2}$ and $\rho _{1}$ are the densities of two fluid layers. In our experiments, $A=0$ as both the layers consist of the same fluid. The growth rates for different measured fluid velocities are estimated using Eq. (1) and tabulated in Table 1. It is found that the growth rate of the KH instability decreases with an increase in the velocity at the interface of two layers of the dust fluid. It essentially means that the growth rate of the KH instability decreases with an increase in the compressibility of the fluid flow.

**Table 1:** Tabulated values of growth rates with Mach number estimated from the Eq. (1).

Inlet gas pressure in Pa	200	300	350	400
velocity difference (*U*) in cm/s	1.8	2.0	2.5	3.0
Mach Number (*M*)	0.83	0.93	1.16	1.40
Growth rate ($\gamma$) in s$^{-1}$	1.13	1.12	0.97	0.34

**Figure 6:**
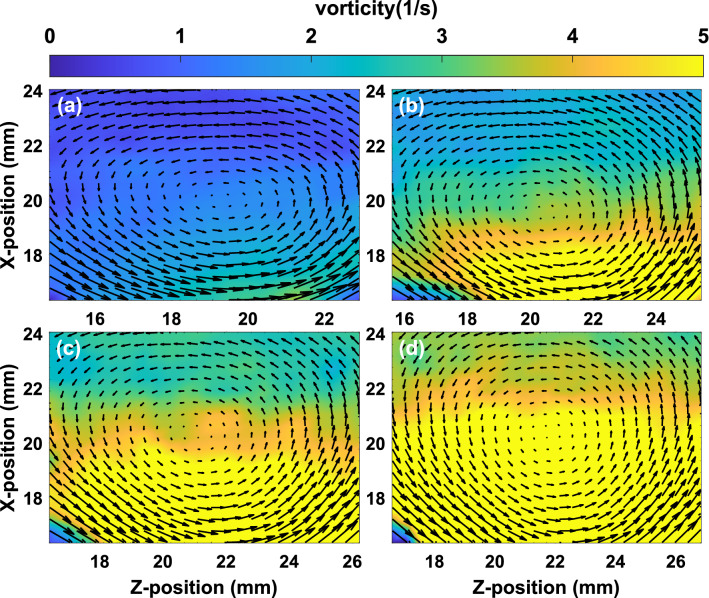
The magnitude of the vorticity ($|\nabla \times v |$) around the vortex at the inlet pressures (**a**) 200 Pa, (**b**) 300 Pa, (**c**) 350 Pa and (**d**) 400 Pa of pulse valve for single layer flow. The arrows on the vorticity plot represent the velocity vectors.

To examine the single mode KH vortex, the vorticity ($\vec {\nabla } \times {\vec v}$) is evaluated over the range of inlet gas pressure of the pulse valve. Fig. 6a–d show its magnitude for the inlet pressures 200 Pa, 300 Pa, 350 Pa, and 400 Pa, respectively. With the increase of gas pressure of the pulse valve, the dust fluid flows with a larger velocity at the interface resulting in the increase of vorticity of the region around the vortex.

**Figure 7:**
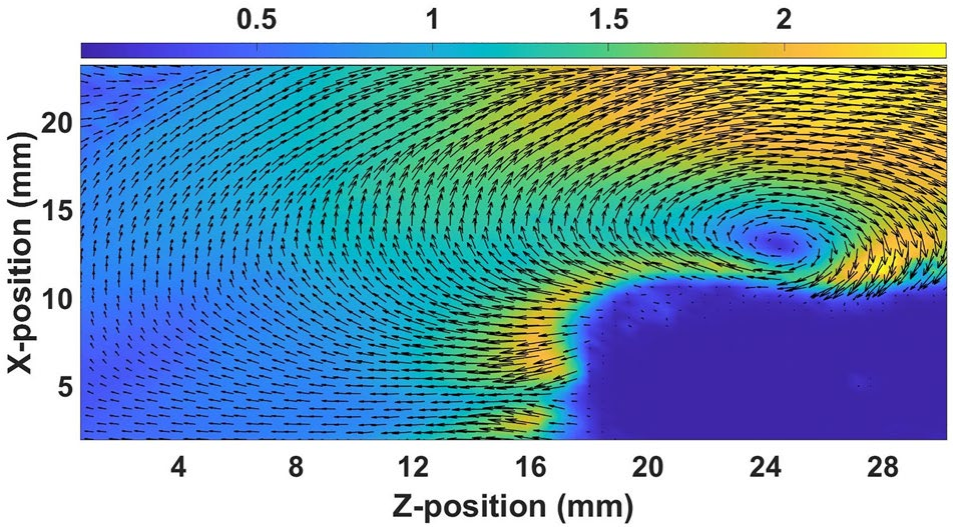
The velocity vector field with the magnitude of the velocity (cm/s) for the double layer flow at the input pressure of the pulse valve is 300 Pa.

To investigate the dynamics of the KH vortex further, the shear velocity at the interface is increased by moving the topmost layer opposite to the bottom layer with the help of single gas injection technique as discussed in Section “Experimental apparatus and procedure”. Fig. 7 shows the color map of the velocity vector field along with its magnitude for the inlet pressure of 300 Pa. In this case, the shear velocity at the interface becomes approximately twofold compared to that of single-layer flow. Due to the increase in shear velocity, the KH vortex appears to be more prominent and smaller in size. In comparison to Fig. 5, the vortex also shifts towards the bottom layer in this situation.

**Figure 8:**
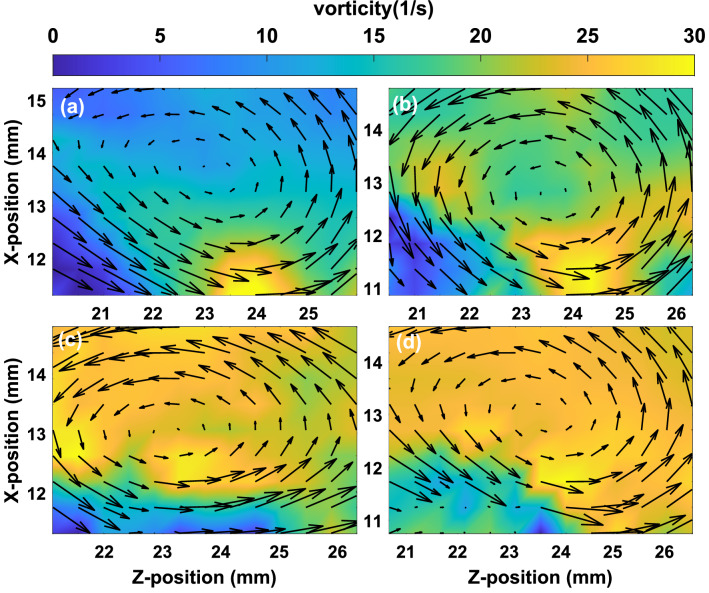
The velocity vectors of the around vortex and the color bar represent the magnitude of vorticity (1/s) at the input pressure (**a**) 200 Pa, (**b**) 300 Pa, (**c**) 350 Pa and (**d**) 400 Pa of pulse valve for two dust layers flow in the opposite direction.

Figure 8 depicts the velocity vectors and the magnitude of vorticity around the vortex for the different input pressures of the gas pulse valve when two layers are made to flow in opposite directions. The magnitude of the vorticity of the vortex at the interface of two oppositely moving dust layers increases with the increase of the inlet pressure of the pulse valve. It is also observed that the vorticity has a larger magnitude in the case of oppositely moving dust layers as compared to the single-layer flow for the same inlet pressure of the pulse valve. It is to be noted that the present investigation is not aimed at studying the impact of interface morphology on flow characteristics. The objective is to study the nature of the KH instability that develops at the interface of a stationary and moving dusty plasma layer. In our experiments, we do not change the manner of generating the flow - only the speed of the flow is changed by varying the injected pressure and the interface remains the same during experiments.

## Molecular dynamic simulation

To provide theoretical support to our experimental observations, Molecular Dynamics (MD) simulations are carried out with the help of an open-source code LAMMPS^60^. The MD simulation is a standard theoretical tool that has been extensively used in the past to analyse and interpret experimental observations in dusty plasmas^40,61–64^. The rationale for this is that MD simulations provide a kinetic level description of the plasma where one tracks the dynamics of individual particles much in the same manner as one does in a dusty plasma experiment where the motion of individual dust particles are video-recorded. The simulations therefore provide a more realistic description of the dust dynamics compared to fluid simulations. For our present simulation, a two-dimensional (2D) periodic system is created containing the charged dust particles, which interact with each other via the Yukawa potential. The parameters used in our simulation are chosen to be the same as the experimental values. The 2D system contains 154,000 charged particles, which are placed in the simulation box of length, $L_{z}=60$ mm and width, $L_{x}=50$ mm. The time step is taken to be 0.001$\omega _{pd}^{-1}$ for the present simulation, where $\omega _{pd} = \sqrt{Q_{d}^{2}/2\pi \varepsilon _{0}m_{d}a^{3}}$ is the characteristic dust plasma frequency. Initially, the 2D system equilibrates by using the Nose-Hoover^65,66^ thermostat by distributing the particles in a canonical (NVT) ensemble. After obtaining the assigned equilibrium temperature, we terminated the canonical thermostat and allowed the system to evolve in the presence of a micro-canonical (NVE) thermostat. This technique makes the system attain a thermodynamic equilibrium at the desired temperature. Likewise, in our experiment, the flow in the bottom layer of the dust fluid is initiated with a velocity over the range of 1.8 cm/s to 3.0 cm/s. In this range of velocities, strong shear is generated at the interface of the stationary and moving dust layers which leads to the formation of a single-mode KH vortex as also observed in the experiments.

**Figure 9:**
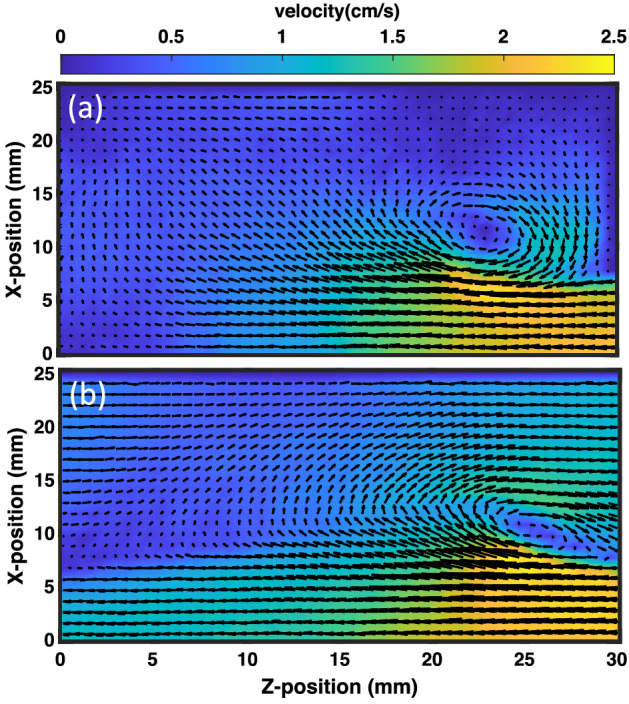
The velocity vector field along with the magnitude of the velocity (cm/s) for (**a**) single-layer flow and (**b**) double-layer flow, which are obtained from the simulation.

Akin to the experiments, the simulation results are initially analyzed by obtaining the velocity vector fields of the flowing dust fluid in the simulation box. In the simulation also the dust fluid is made to flow in one direction (single-layer flow) as well as in opposite directions (double-layer flow). Fig. 9a depicts the velocity vector field along with its magnitude when the fluid is made to flow with a velocity of 2.0 cm/s. Due to this directional velocity of the bottom layer, a shear is generated at the interface which leads to the formation of a vortex in the dust fluid at a location of Z = 23 mm and X = 11 mm. In the second case, the bottom and top layers of charged particles are made to flow in opposite directions with a velocity of 2 cm/s and 1 cm/s, respectively to increase the shear velocity at the interface of the dust fluid. Fig. 9b depicts the velocity vector field along with the magnitude of the velocity in this situation. It is to be noted that the KH vortex also forms at Z = 25 mm and X = 11 mm in the case of double-layer flows. However in this case the vortex becomes elongated. These simulation findings for single and double-layer flows closely resemble the experimental observations of the formation of KH vortex.

**Figure 10:**
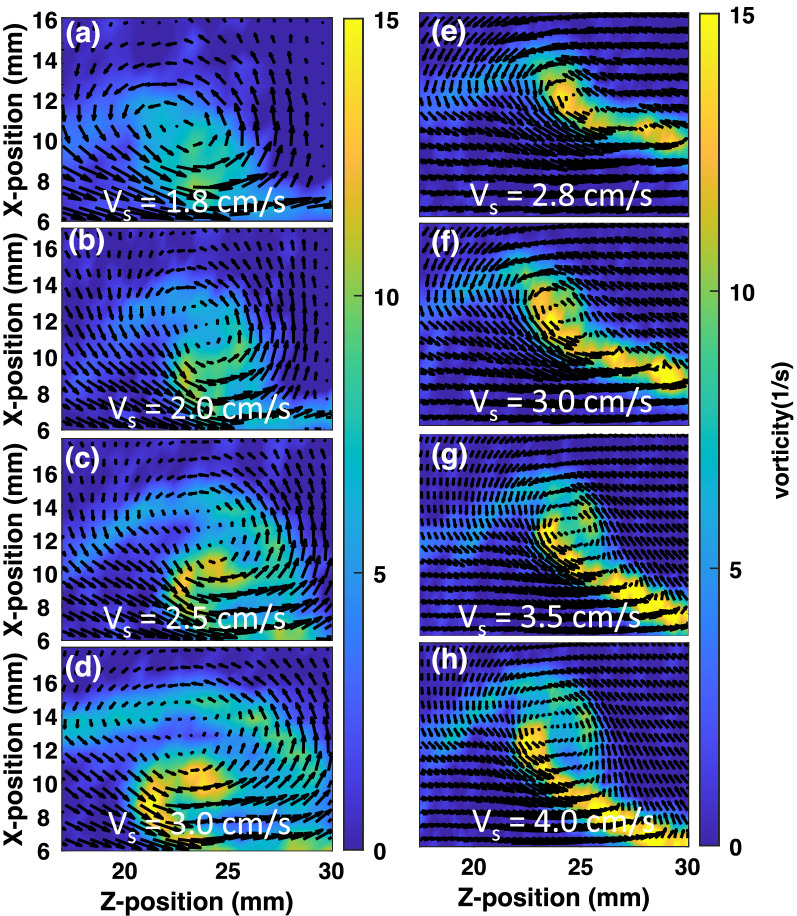
The velocity vectors around the vortex structure and the color map represent the magnitude of vorticity (1/s) for the single-layer flow of shear velocities of (**a**) 1.8 cm/s, (**b**) 2.0 cm/s, (**c**) 2.5 cm/s, (**d**) 3.0 cm/s and double-layer flow of shear velocities of (**e**) 2.8 cm/s, (**f**) 3.0 cm/s, (**g**) 3.5 cm/s and (**h**) 4.0 cm/s, which are obtained from the simulation.

The vorticity around the vortex structure is also estimated using the simulation data for different values of fluid flow in the case of single and double-layer flows. Fig. 10 shows the color map of the magnitude of the vorticity around the vortex region along with the velocity vector for the different values of shear velocities ($V_s$) for the single layer flow (left panel) and double layer flow (right panel). It is observed that the vorticity of the vortex increases with the increase in the velocity of the dust layer for both cases.

**Figure 11:**
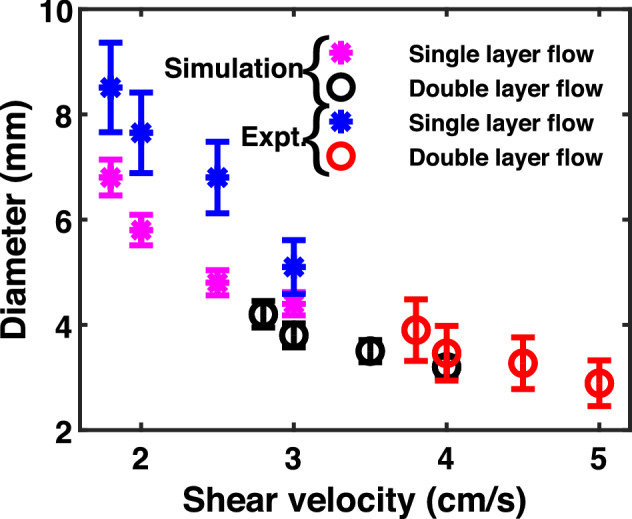
Variation of the diameter of the KH vortex for single layer flow (*) and double layer flow (O) with the shear velocity.

For a quantitative analysis, the average diameter of the KH vortex is measured over a range of shear velocities as shown in Fig. 11. The simulation data points with ‘*’ (magenta) represent the diameter of the KH vortex when the single dust layer is made to flow, whereas the data points associated with ‘o’ (black) indicate the diameter of the vortex when the flow in the stationary dust layer is initiated in the opposite direction with a higher shear velocity. It is clear from the figure that the diameter of the KH vortex decreases with the increase in the shear velocity at the interface of the two dust fluid layers.

The simulation results are also compared with the experimentally obtained vortex diameter for a range of shear velocities. The experimental data points with ‘*’ (blue) represent the diameter of the vortex for the single dust layer flow, whereas the data points with ‘o’ (red) indicate the diameter of the vortex for the double layer flow. Interestingly, the experimental results follow the same trend of vortex size as the simulation findings. However, the vortex size becomes always bigger in the experiments in comparison to the simulation. This may be due to the neglect, in the MD simulations, of experimental effects like the effects of dust-neutral collisions, influence of the background plasma, finite boundary effects *etc.*.

## Conclusion

To conclude, an experimental demonstration of the single-mode Kelvin-Helmholtz instability is presented for the first time in a compressible dust fluid flow. The experiments are carried out in the Dusty Plasma Experimental (DPEx) device, and the dusty plasma is produced in a DC glow discharge Argon plasma environment using micron-sized Kaolin particles. A gas pulse valve is employed to make compressible flow in a particular layer of this dust fluid. A strong shear is generated at the interface of the moving and the stationary layers of the dust fluid. This leads to the emergence of a single mode Kelvin-Helmholtz instability that nonlinearly produces a vortex structure at the interface. It is found that the growth rate of the instability decreases as the interface velocity of the two layers of the dust fluid increases. To increase the shear velocity, the stationary layer is also made to flow in opposite directions. It is found that the vorticity of the vortex increases with the increase in the shear velocity in the dust fluid. It is observed that the diameter of the vortex decreases with the increase in the shear velocity of the dust layer. To simulate our experiments, we have performed a molecular dynamics simulation with the open-source code LAMMPS. An equilibrium system of the charged particles is created with the same experimental parameters. Similar to our experiments, a directional motion to a layer of charged particles is provided with the same velocity which is measured in the experiments. The results obtained from molecular dynamic simulation are found to be in good agreement with our experimental observations. Our investigations, obtained under controlled laboratory conditions, can help in the understanding of the formation of Kelvin-Helmholtz instability in astrophysical jets and protoplanetary disks, which have a compressible flow of dense ionised gas along with charged dust particles.

## Data Availability

The datasets used and/or analysed during the current study available from the corresponding author on reasonable request.
